# Dysregulation of Ubiquitin-Proteasome System in Neurodegenerative Diseases

**DOI:** 10.3389/fnagi.2016.00303

**Published:** 2016-12-15

**Authors:** Qiuyang Zheng, Timothy Huang, Lishan Zhang, Ying Zhou, Hong Luo, Huaxi Xu, Xin Wang

**Affiliations:** ^1^Fujian Provincial Key Laboratory of Neurodegenerative Disease and Aging Research, Institute of Neuroscience, College of Medicine, Collaborative Innovation Center for Brain Science, Xiamen UniversityXiamen, China; ^2^Neuroscience Initiative, Sanford Burnham Prebys Medical Discovery Institute, La Jolla, CAUSA

**Keywords:** ubiquitin, deubiquitinating enzyme, proteasome, Alzheimer’s disease, Parkinson’s disease, Huntington’s disease

## Abstract

The ubiquitin-proteasome system (UPS) is one of the major protein degradation pathways, where abnormal UPS function has been observed in cancer and neurological diseases. Many neurodegenerative diseases share a common pathological feature, namely intracellular ubiquitin-positive inclusions formed by aggregate-prone neurotoxic proteins. This suggests that dysfunction of the UPS in neurodegenerative diseases contributes to the accumulation of neurotoxic proteins and to instigate neurodegeneration. Here, we review recent findings describing various aspects of UPS dysregulation in neurodegenerative disorders such as Alzheimer’s disease, Parkinson’s disease, and Huntington’s disease.

## Abnormal Protein Aggregation in Neurodegenerative Diseases

Many neurodegenerative diseases are associated with aggregate-prone neurotoxic proteins (aggresomes or inclusion bodies) that perturb cellular homeostasis and neuronal function (Popovic et al., 2014). These diseases are also categorically classed as proteinopathies, and comprise aggregation-prone components such as β-amyloid (Aβ) and tau in Alzheimer’s disease (AD), α-synuclein in Parkinson’s disease (PD), and polyglutamine (polyQ) repeat diseases.

Alzheimer’s disease is the most common form of progressive neurodegeneration and represents the main cause of senile dementia affecting about 10% of the population over the age of 65 and about 50% of the population over 85. AD can be divided into two forms: familial AD and sporadic AD. Familial AD accounts for 5%∼10% of AD patients, where inherited autosomal dominant mutations in three genes have been considered as the primary causes of familial AD: these include mutations in the genes encoding β-amyloid precursor protein (APP) and presenilins (PS1 and PS2; Goate et al., 1991; Levy-Lahad et al., 1995; Sherrington et al., 1995). In addition, sporadic AD is associated with polymorphisms in the Apolipoprotein E (APOE) gene, where the epsilon four allele is a strong genetic risk factor for late-onset AD (Strittmatter et al., 1993; Bu, 2009; Fu et al., 2016). Accumulation and aggregation of neurotoxic proteins, such as β-amyloid (Aβ), hyperphosphorylated tau, ubiquitinated proteins, and other unfolded proteins in vulnerable brain regions such as the hippocampus and cortex in AD brain is central to disease pathogenesis (Selkoe, 2001). Two major pathological hallmarks in AD are extracellular amyloid plaques and intraneuronal neurofibrillary tangle (NFT; Zhang et al., 2011). NFTs are comprised of hyperphosphorylated microtubule-associated protein tau (Song et al., 2015; Yamada et al., 2015; Zempel and Mandelkow, 2015). Amyloid plaques are primarily comprised of Aβ which is derived from sequential cleavages of APP by β- and γ-secretases (Zhang et al., 2011; Audrain et al., 2016). Cumulative evidence demonstrates that Aβ and tau are neurotoxic and can trigger a cascade of neurodegenerative processes ending in neuronal death, suggesting that overproduction/accumulation of Aβ and tau in vulnerable brain regions is the primary influence driving AD pathogenesis (Hardy and Selkoe, 2002; Gautam et al., 2015; Golovyashkina et al., 2015; Popugaeva et al., 2015).

Parkinson’s disease is the second most common neurodegenerative disease, clinically characterized by motor abnormalities, including resting tremor, rigidity, hypokinesia, and postural instability (Dauer and Przedborski, 2003; More and Choi, 2015). The loss of dopaminergic neurons in the substantial nigra subregion of midbrain is the major cause of motor deficits (Chen et al., 2015). Major pathological features of PD include intraneuronal accumulation of Lewy bodies and dystrophic neurites (Lewy neurites), both of which comprise aggregated proteins, such as α-synuclein, parkin, and ubiquitinated proteins (Hughes et al., 1992; Braak et al., 2003; Chiasserini et al., 2015; Xiang et al., 2015). Sporadic PD accounts for more than 90% of PD cases (Lang and Lozano, 1998), and only less than 10% of familial PD cases are caused by monogenic mutations (Benitez et al., 2016). Mutations in the gene encoding α-synuclein (Polymeropoulos et al., 1997; Helferich et al., 2015; Majbour et al., 2016; Schonherr et al., 2016) represent autosomal dominant familial PD, and mutations in genes encoding parkin (Kitada et al., 1998; Cha et al., 2015), UCHL1 (Leroy et al., 1998; Maraganore et al., 1999), DJ-1 (Bonifati et al., 2003), PINK1 (Valente et al., 2004), and LRRK2 (Zimprich et al., 2004; Saha et al., 2015) cause autosomal recessive familial PD.

Huntington’s disease (HD) is an autosomal dominant progressive neurodegenerative disorder, clinically characterized by cognitive impairment, chorea and dystonia, bradykinesia, uncoordinated movement, mood disorder and other psychiatric symptoms, and behavioral difficulties (Huntington Study Group, 1996). As the most common of the polyQ disorders, HD is caused by expansion of a CAG repeat coding for polyQ in the N-terminus of the huntingtin protein (MacDonald et al., 1993; Evers et al., 2015; Nekrasov et al., 2016). There is a remarkable repeat length threshold effect, where polyQ chain expansion of 36 repeats triggers HD onset, whereas repeats of less than 35 are non-pathological (Chong et al., 1997). The polyQ expansion in the N-terminus of huntingtin protein confers a gain-of-function phenotype, and is associated with significant neurotoxicity (Tallaksen-Greene et al., 2003).

In addition to huntingtin, expansion of polyQ-encoding CAG repeats in several genes have also been found in other polyQ repeat diseases, such as Ataxin-1 in spinocerebellar ataxia type 1 (SCA1), Ataxin-2 in SCA2, Atantin-3 in SCA3, α1_A_-voltage-dependent calcium channel (CACNA1A) in SCA6, Ataxin-7 in SCA7, Atrophin-1 in dentatorubral-pallidoluysian atrophy (DRPLA), and androgen receptor in SBMA (Zoghbi and Orr, 2000; Del Cano-Espinel et al., 2015). All polyQ repeat disorders are dominant neurodegenerative diseases and are associated with pathological intracellular inclusion bodies in the affected brain regions (Ross et al., 1997; Zoghbi and Orr, 2000).

## Ubiquitin-Proteasome System

Ubiquitin is an evolutionarily conserved 76-amino acid moiety covalently tandemly linked to target protein components for degradation by the ubiquitin-proteasome system (UPS), and is required for degradation of about 80% intracellular proteins in eukaryotes (Pickart, 2001). The presence of ubiquitin in intracellular inclusions has been found in various neurodegenerative diseases, for example, amyloid plaques and NFT in AD, Lewy bodies in PD, and intranuclear inclusions in polyQ disorders (Todi and Paulson, 2011).

**FIGURE 1:**
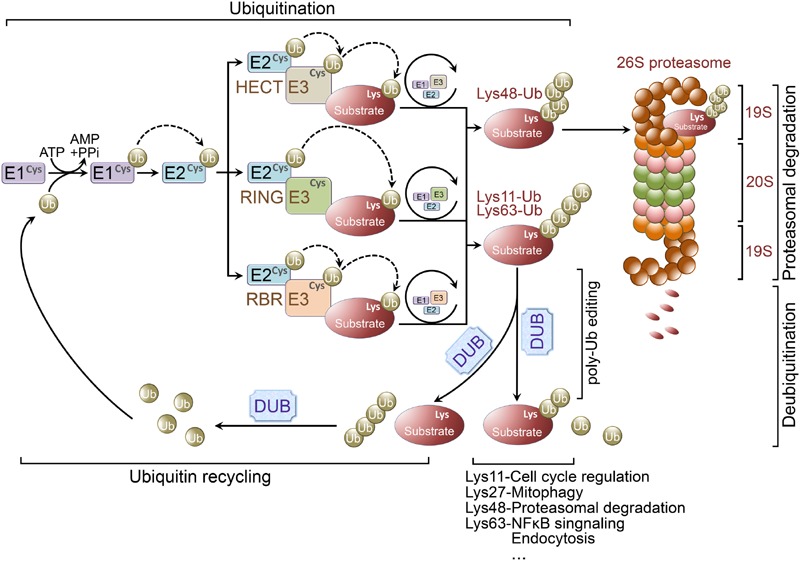
**Ubiquitination and ubiquitin-proteasome system.** Ubiquitination is a posttranslational modification that the ubiquitin is covalently conjugated to a Lysine residue of the substrate proteins. Ubiquitin is first attached to a Cysteine residue (the active-site) of ubiquitin activating enzyme (E1 ligase) in an ATP-dependent reaction. Subsequently, the activated ubiquitin will be transferred to a Cysteine residue of conjugating enzyme (E2 ligase). Finally, working with a specific E3 ligase, E2 ligase can transfer the (poly)ubiquitin to a Lysine residue of the substrate. There are three major classes of E3 ligases, including RING E3 ligases, HECT E3 ligases, and RBR E3 ligases. RING E3 ligases catalyze the transfer of ubiquitin directly from E2 ligases to the substrate. However, the activated ubiquitin is first transferred from E2 ligases to HECT or RBR E3 ligases and then be transferred to the substrate from the E3 ligases. Lys48-linked polyubiquitin chains usually target proteins for proteasomal degradation, whereas Lys63-linked polyubiquitination is involved in regulation of NFκB signaling. Lys27-linked polyubiquitination is important in regulating mitophagy, and Lys11-linked polyubiquitin chains are implicated in cell cycle regulation. Ubiquitination is reversed by deubiquitinating enzymes (DUBs). DUBs are crucial for determining the fate of ubiquitinated proteins through removing/editing the length/type of polyubiquitin chains. In addition, DUBs can disassemble unanchored ubiquitin chains for ubiquitin recycling.

Protein ubiquitination occurs through the coordinated activity of several enzymes, including an ubiquitin activating enzyme (E1 ligase), conjugating enzyme (E2 ligase), and E3 ligase. Initially, a single ubiquitin moiety is attached to an active-site Cysteine residue within the E1 ligase through a thioester bond in an ATP dependent manner. The activated ubiquitin is then transferred to an ubiquitin conjugating enzyme-E2 ligase. Finally, the E2 ligase will cooperatively transfer the ubiquitin chain with a specified E3 ligase to a particular substrate, whereby the 26S proteasome will target the polyubiquitinated protein for degradation (**Figure 1**).

Ubiquitin E3 ligases determine the specificity of ubiquitination process. E3 ligases are grouped into three classes according to their unique domains, including Really Interesting New Gene (RING) or U-box domain-containing E3 ligases, Homologous to E6AP C-terminus (HECT) domain-containing E3 ligases, and RING-between-RING (RBR) domain-containing E3 ligases (Atkin and Paulson, 2014; Morreale and Walden, 2016). RING E3 ligases catalyze the transfer of ubiquitin directly from E2 ligases to the substrates by binding both of them. Whereas HECT E3 ligases comprise a conserved Cysteine residue as an ubiquitin acceptor, where the activated ubiquitin is initially transferred from the E2 ligases to HECT E3 ligases prior to E3-mediated substrate conjugation (Ravid and Hochstrasser, 2008). Unlike RING E3 ligases, RBR E3 ligases contain two RING domains (RING1 and RING2) separated by an in-between-RING (IBR) domain. Functionally, RBR E3 ligases are similar with HECT E3 ligases, both catalyze ubiquitin first transfer from E2 ligases to a catalytic Cysteine residue on E3 ligases then from the E3 ligases to the substrates (Spratt et al., 2014) (**Figure 1**).

The fate of an ubiquitin-linked protein is dependent on Lysine chain-specific polyubiquitination (Grabbe et al., 2011; Kulathu and Komander, 2012; Rieser et al., 2013). For example, Lys48-linked polyubiquitin chains usually target proteins for degradation (Husnjak et al., 2008), whereas Lys63-linked polyubiquitination is involved in regulation of NFκB signaling (Laplantine et al., 2009), Lys27-linked polyubiquitin chains are important for mitophagy (Geisler et al., 2010), and Lys11-linked polyubiquitination is implicated in cell cycle regulation (Bremm and Komander, 2011). Therefore, ubiquitination is not only important for protein degradation, but is also implicated in non-proteolytic functions, such as endocytosis, protein-protein interaction, intracellular trafficking, inflammatory signaling, autophagy, and DNA repair (Grabbe et al., 2011; Kulathu and Komander, 2012; Rieser et al., 2013).

The UPS is involved in protein quality control and removal of misfolded and aggregated proteins, and dysfunction of the UPS is implicated in the pathogenesis of neurodegenerative diseases (Popovic et al., 2014).

## Deubiquitinating Enzymes

The process of ubiquitination is reversible and the reverse process is called deubiquitination which is accomplished by deubiquitinating enzymes (DUBs; Rubinsztein, 2006; Reyes-Turcu et al., 2009; Popovic et al., 2014). Increasing evidence indicates the importance of DUBs in regulating UPS function, including proofreading ubiquitin-protein conjugates, removing ubiquitin from conjugated proteins as key examples (Amerik and Hochstrasser, 2004) (**Figure 1**). Pivotal roles for DUBs have been demonstrated in maintaining neuronal functions and regulating the pathogenesis of various neurodegenerative diseases (Rubinsztein, 2006; Todi and Paulson, 2011; Popovic et al., 2014).

More than 100 DUBs have been identified (Popovic et al., 2014), and can be subdivided into five subfamilies. DUBs can be further categorized into two classes, the Cysteine proteases and the zinc-dependent metalloproteases (Todi and Paulson, 2011). Cysteine protease-associated DUBs include the ubiquitin carboxyl-terminal hydrolases (UCHs), ubiquitin specific proteases (USPs), Machado Joseph Diseases (MJD) proteases, and ovarian tumor (OTU) protease. Metalloprotease DUBs comprise the JAB1/MPN/Mov34 metalloenzyme (JAMM) subfamily.

UCHL1, a member of the UCH subfamily, is one of the most abundant proteins in the brain. UCHL1 is known to interact with and stabilize monomeric ubiquitin, and facilitates the E3 ligase activity in its dimeric form (Doran et al., 1983; Wilkinson et al., 1989). An *Aplysia* UCHL1 ortholog, ubiquitin carboxyl-terminal hydrolase (AP-Uch) has been found to be important in maintaining synaptic function (Hegde et al., 1997). Inhibiting the expression or function of AP-Uch blocks induction of long-term facilitation, which is thought to be the molecular basis for learning and memory (Hegde et al., 1997). Mammalian UCHL1 is required for normal synaptic structure and function in hippocampal neurons (Cartier et al., 2009). Activation of NMDA receptor upregulates UCHL1 activity and the levels of free ubiquitin monomers. Inhibition of UCHL1 disrupts distribution of synaptic proteins, increases dendritic spine size and reduces spine density. However, restoration of ubiquitin in UCHL1-inhibited neurons rescues impaired synaptic structure (Cartier et al., 2009). UCHL1 is also essential for normal structure and function of neuromuscular junctions (NMJs), reduction of synaptic vesicles and accumulation of tubulovesicular structures at the presynaptic nerve terminals have been observed in *Uchl1* knockout mice (Chen et al., 2010). UCHL3, a homolog of UCHL1, is involved in spatial and working memory (Wood et al., 2005).

19S proteasome-associated DUBs comprise three members: USP14, UCH37, and RPN11, which show differences in ubiquitin chains removals and consequent protein degradation (Lee et al., 2011). It has been reported that both USP14 and UCH37 prevent substrate degradation by removing ubiquitin chains and promoting proteasomal substrate dissociation. In contrast, RPN11 cleaves at the base of the ubiquitin-linked substrates and promotes substrate degradation (Lee et al., 2011). USP14 is essential for synaptic development and normal function of NMJs (Chen et al., 2009), which is mediated through ubiquitination and activation of c-Jun N-terminal kinase signaling (Vaden et al., 2015). Pharmacological inhibition of USP14 enhances proteasomal degradation of several neurotoxic proteins, including tau, TDP14, and Ataxin-3 (Lee et al., 2010). *Usp14*-deficient mice display motor neuron deficits and developmental NMJ defects (Chen et al., 2009). USP16, a chromosome 21-encoded gene, is involved in chromatin remodeling and cell cycle progression through regulated ubiquitination of histone H2A (Joo et al., 2007). USP16 interacts with HERC2 and modulates the ubiquitination in DNA repair machinery components (Zhang et al., 2014). USP16 is upregulated in Down’s syndrome (DS) cells due to extrachromosomal triplication in trisomy 21, downregulation of USP16 partially restores the impaired proliferation in DS somatic stem cells (Adorno et al., 2013). It has been reported that USP16 acts as a H2A DUB and regulates hematopoiesis and hematopoietic stem cell function (Gu et al., 2016). Moreover, USP16 is required for embryonic stem cell differentiation, where USP16 deficiency leads to embryonic lethality in mouse deletion models (Yang et al., 2014).

The gene encoding USP25 also resides on human chromosome 21 (Valero et al., 1999), USP25 is involved in protein degradation by the 26S proteasome (Valero et al., 1999). USP25 is also implicated in inflammation, and it has been found to facilitate removal of Lys63-linked ubiquitin chains from tumor necrosis factor-receptor associated factor 5/6 (TRAF5/6), *Usp25*-deficient mice show higher sensitivity to IL-17 dependent inflammation and autoimmunity (Zhong et al., 2012).

## Dysregulation of Ubiquitination in Neurodegenerative Diseases

Accumulating evidence indicates that dysfunction of the UPS is a key factor to initiate and aggravate the pathogenesis of neurodegenerative diseases. We will focus on the dysregulation of ubiquitination and proteasomal degradation in neurodegenerative pathology in AD, PD, and HD (**Table 1**).

**Table 1:** Ubiquitin-proteasome system is implicated in neurodegenerative diseases pathogenesis.

Disease	Protein	Disease Relevance	Pathogenesis	Reference
AD	Parkin (E3 ligase)	Decreased in AD	Parkin overexpression reduces Aβ and restores impaired LTP and behavioral abnormalities of APP/PS1 mouse model.	Rosen et al., 2010; Hong et al., 2014
AD	UCHL1 (DUB)	Decreased in AD	UCHL1 overexpression improves contextual memory and restores synaptic functions of APP/PS1 mouse model.	Choi et al., 2004; Gong et al., 2006
AD	HRD1 (E3 ligase)	Decreased in AD	HRD1 reduces Aβ generation through interacting with APP and facilitating APP ubiquitination and proteasomal degradation.	Kaneko et al., 2010
AD	CHIP (E3 ligase)	Upregulated in AD	CHIP ubiquitinates phosphorylated tau. Deletion of CHIP leads to the accumulation of non-aggregated, hyperphosphorylated, as well as caspase-3-cleaved tau species. Overexpression of CHIP and Hsp70/90 decreases the steady-state Aβ levels and promotes Aβ degradation.	Petrucelli et al., 2004; Shimura et al., 2004; Dickey et al., 2006, 2007; Kumar et al., 2007
PD	Parkin (E3 ligase)	Decreased in PD with loss-of-function mutations	More than 100 different *PARK2* (encoding parkin) mutations have been found in PD patients, including missense, truncation, copy number variations, deletions, and insertions. Loss-of-function mutations in parkin impair its E3 ligase activity and lead to the accumulation of α-synuclein and the formation of Lewy bodies.	Shimura et al., 2001; Lesage and Brice, 2009; Lu et al., 2009; Chung et al., 2013
PD	UCHL1 (DUB)	Mutation on I93M and S18Y.	UCHL1^I93M^ mutation promotes its dimerization and inhibits the proteasomal degradation of α-synuclein through the Lys63-linked polyubiquitination of α-synuclein.	Leroy et al., 1998; Liu et al., 2002; Maraganore et al., 2004; Setsuie et al., 2007
PD	CHIP (E3 ligase)	NA	CHIP can enhance parkin’s E3 ligase activity. Moreover, CHIP ubiquitinates LRRK2 and α-synuclein and promotes their degradation.	Imai et al., 2002; Ko et al., 2009; Kalia et al., 2011
PD	TRAF6 (E3 ligase)	Upregulated in PD	TRAF6 promotes Lys6-, Lys27-, and Lys29-linked ubiquitination of DJ-1 and α-synuclein. TRAF6 induces Lys63-linked ubiquitination of PINK1, and stabilizes PINK1 on the depolarized mitochondria.	Zucchelli et al., 2010; Chung et al., 2013; Murata et al., 2013
HD	CHIP (E3 ligase)	NA	CHIP suppresses aggregation and toxicity of polyQ-huntingtin. Knockdown of CHIP in HD transgenic mice aggravates disease pathology.	Miller et al., 2005
SCA3	Ataxin-3 (E3 ligase)	Gain-of-function mutation with polyQ expansion	Ataxin-3 overexpression protects *Drosophila* from polyQ-huntingtin-induced neurodegeneration. MJD-linked Ataxin-3 mutations promote ubiquitination and autophagic degradation of parkin.	Warrick et al., 2005; Durcan et al., 2011

### Dysregulation of the UPS in Alzheimer’s Disease

The presence of ubiquitin in NFTs and amyloid plaques in AD brain has been described as early as 1987 (Cole and Timiras, 1987; Mori et al., 1987; Perry et al., 1987). Ubiquitin-targeted proteasomal activity declines with age Tg2576 AD mouse model brain, and Aβ treatment markedly attenuates proteasomal activity in cultured neurons (Oh et al., 2005). Aβ induced UPS dysfunction contributes to the accumulation of reticulon 3 (RTN3) in dystrophic neurites, and the presence of dystrophic neurites and clustering of tubular endoplasmic reticulum has been observed in RTN3 transgenic mouse brain (Hu et al., 2007; Sharoar et al., 2016). Interestingly, intracellular Aβ accumulation and impaired proteasome function can be reversed by the ubiquitin E3 ligase parkin (Rosen et al., 2010). Parkin expression is downregulated in AD brains, and reduced parkin expression may contribute to the accumulation of intracellular Aβ. Moreover, parkin expression results in Aβ reduction which can be dampened by proteasome inhibitors (Rosen et al., 2010). In addition, overexpression of parkin in APP/PS1 mouse models restores impaired long-term potentiation (LTP) and rescues behavioral abnormalities by reducing Aβ load and neuroinflammation (Hong et al., 2014).

UCHL1 has been observed in ubiquitin-enriched inclusion bodies in AD brains (Lowe et al., 1990). In addition, decreased soluble UCHL1 protein levels was detected in the brains of postmortem AD patients and APP/PS1 mouse models (Choi et al., 2004; Gong et al., 2006), and soluble UCHL1 is inversely proportional to the number of NFTs in AD brains (Choi et al., 2004). Upregulation of UCHL1 improves contextual learning and restores synaptic functions in APP/PS1 mouse models, where rescue effects are dependent on enzymatic UCHL1 activity (Gong et al., 2006). Moreover, overexpression of UCHL1 reduces the levels of β-secretase BACE1, and consequent BACE1 cleavage products (APP C-terminal fragment C99 and Aβ). Depletion of UCHL1 increases the levels of BACE1, C99, and Aβ in the *Uchl1*-null gad mice (Zhang et al., 2012).

A direct interaction between APP and ubiquitin ligases also contributes to amyloid pathology. HRD1, an endoplasmic reticulum-associated degradation (ERAD) ubiquitin E3 ligase, is reduced in AD brains (Kaneko et al., 2010). HRD1 interacts with APP through its Proline-rich region, and this interaction reduces Aβ generation through promoting APP ubiquitination and degradation (Kaneko et al., 2010). In addition, APP can bind to ubiquitin E3 ligases Stub1 and CRL4^CRBN^ through the APP cytosolic region (ACR; Del Prete et al., 2016). ACR promotes the ubiquitination of several presynaptic proteins and regulates neurotransmitter release (Del Prete et al., 2016).

Polyubiquitinated tau has also been found in the brains of AD patients (Perry et al., 1987), where ubiquitin conjugation usually occurs at Lys254, Lys311, and Lys353 within the tau microtubule-binding domain (Cripps et al., 2006). In AD brains, accumulation of hyperphosphorylated, ubiquitinated tau protein has been found at both presynaptic and postsynaptic terminals, which is associated with dysfunction of the UPS (Tai et al., 2012). Recently, Myeku et al. reported that tau can induce dysfunction of the 26S proteasome which can be prevented by activating cAMP-PKA signaling. Protective effects derived from cAMP-PKA signaling are mediated through phosphorylation of the 26S proteasomal subunit (Myeku et al., 2016). Han et al. directly delivered purified proteasomes into cells through mesoporous silica nanoparticle-mediated endocytosis, and they found that extraproteasomal transfer reduces tau aggregates and promotes cell survival against tau-induced proteotoxic stress (Han et al., 2014). C-terminus of Hsp70-interacting protein (CHIP), an ubiquitin E3 ligase, is also implicated in AD pathogenesis. It has been found that both CHIP and Hsp70 are upregulated in the brains of AD patients (Sahara et al., 2005). CHIP and its binding protein Hsp70 can induce the ubiquitination and degradation of phosphorylated tau (Petrucelli et al., 2004; Shimura et al., 2004), deletion of CHIP leads to the accumulation of non-aggregated, hyperphosphorylated, as well as caspase-3-cleaved tau (Dickey et al., 2006). Moreover, inhibition of Hsp90 reduces hyperphosphorylated tau through an interaction with CHIP (Dickey et al., 2007). In addition, overexpression of CHIP and Hsp70/90 decreases steady-state Aβ levels and promotes Aβ degradation (Kumar et al., 2007).

### Dysregulation of UPS in Parkinson’s Disease

The presence of ubiquitin-immunopositive Lewy bodies in the brains of PD patients has been observed about 30 years ago (Kuzuhara et al., 1988). Impaired proteasomal function in the substantia nigra of PD brains was later observed (McNaught and Jenner, 2001). Importantly, genetic evidence reveals a clear involvement of UPS dysfunction in PD pathogenesis: familial PD-associated genetic variations have been identified on *PARK2* (Kitada et al., 1998) and *UCHL1* (Leroy et al., 1998; Maraganore et al., 1999), which encode the ubiquitin E3 ligase parkin and deubiquitinating enzyme UCHL1, respectively. Recessive early-onset familial PD can also be caused by mutations in genes encoding DJ-1 (Abou-Sleiman et al., 2003; Bonifati et al., 2003) and PINK1 (Valente et al., 2004), presumably through a loss-of-function mechanism.

An interaction between parkin and its substrate α-synuclein is a crucial regulatory component of dopaminergic neuron degeneration, loss-of-function mutations in *PARK2* usually cause early-onset autosomal recessive PD (Kitada et al., 1998), whereas disease-associated mutations in α-synuclein are usually associated with autosomal dominant PD (Polymeropoulos et al., 1997). Mutant parkin fails to interact with α-synuclein, leads to the accumulation of α-synuclein and the formation of Lewy bodies (Shimura et al., 2001). Transgenic mice carrying mutant parkin^Q311X^ develop progressive hypokinetic motor deficits, accumulation of α-synuclein and age-dependent loss of dopaminergic neurons in the substantia nigra and striatum (Lu et al., 2009).

Parkin is an E3 ligase mediating multiple forms of ubiquitination, including monoubiquitination, Lys48- and Lys63-linked polyubiquitination (Moore, 2006). In addition, parkin can form an E3 ligase complex with PINK1 and DJ-1 to promote unfolded protein degradation (Xiong et al., 2009): PD mutations in each component impair E3 ligase activity (Xiong et al., 2009). CHIP can promote parkin-mediated ubiquitination of Pael receptor and inhibit the Pael receptor-induced cell death (Imai et al., 2002). In addition, CHIP ubiquitinates and facilitates proteasomal degradation of LRRK2, where knockdown of CHIP exacerbates neurotoxicity mediated by mutant LRRK2 (Ko et al., 2009). CHIP can ubiquitinate α-synuclein and reduce toxic α-synuclein oligomers (Kalia et al., 2011). TRAF6 is upregulated in the brains of PD patients (Chung et al., 2013), and TRAF6 promotes Lys6-, Lys27-, and Lys29-linked ubiquitination of DJ-1 and α-synuclein. This interaction may cause aggregation of insoluble and polyubiquitinated mutant DJ-1 proteins (Zucchelli et al., 2010). Moreover, TRAF6 ubiquitinates PINK1 at Lys433 residue as a poly-Lys63 conjugated form which stabilizes PINK1 in depolarized mitochondria. Down-regulation of TRAF6 disrupts PINK1 localization, thereby causing mitochondria damage through parkin recruitment (Murata et al., 2013). Mutations in *UCHL1* have been reported in rare cases of early-onset familial PD (Leroy et al., 1998; Maraganore et al., 1999). Most notably, the UCHL1^I93M^ PD variant shows reduced deubiquitinating activity (Leroy et al., 1998). Ubiquitin-positive inclusion bodies and the loss of dopaminergic neurons have been observed in the substantia nigra of UCHL^I93M^ transgenic mouse brains (Setsuie et al., 2007). In addition, UCHL1^I93M^ mutation promotes dimerization and Lys63-linked polyubiquitination of α-synuclein. Lys63-linked polyubiquitination may aggravate PD pathology by inhibiting proteasomal degradation of α-synuclein (Liu et al., 2002).

### Dysregulation of UPS in Huntington’s Disease

HD patients manifest polyQ repeats of over 40 residues within the N-terminus of huntingtin protein. Ubiquitin-linked mutant huntingtin has been found in neuronal intranuclear inclusions and dystrophic neurites in the cortex and striatum of HD patients (Difiglia et al., 1997). However, it seems that eukaryotic proteasomes fail to cleave and eliminate pathogenic protein species with repeats greater than 25Q residues; this results in accumulation and aggregation of huntingtin protein in the neuronal nucleus (Venkatraman et al., 2004). However, it has been reported that a selective histone deacetylase (HDAC) inhibitor 4b ameliorates cognitive impairment in N171-82Q HD mouse model by preventing formation of nuclear huntingtin aggregates. Mechanistically, HDAC inhibition upregulates expression of multiple genes that are fundamental to protein phosphorylation and ubiquitination, including *Ube2K, Ubqln, Ube2e3, Usp28, Sumo2*, as well as the genes encoding components of the inhibitor of κB kinase (IKK) complex (Jia et al., 2012). It has been reported that CHIP suppresses polyQ aggregation and toxicity, and knockdown of CHIP in HD transgenic mice aggravated disease pathology (Miller et al., 2005).

Spinocerebellar ataxia type 3 (SCA3) is another polyQ repeat disease, which is caused by a polyQ-encoding CAG repeat expansion in the gene encoding Ataxin-3 (Kawaguchi et al., 1994). SCA3 is characterized by progressive ataxia with degeneration in the cerebellum, brain stem, substantia nigra, and globus pallidus interna. Clinical symptoms of SCA3 include impaired eye movement, speech and swallowing defects (Todi and Paulson, 2011). Ataxin-3 is a DUB from MJD subfamily of proteases which contain an ubiquitin interacting motif flanking the polyQ domain. Ataxin-3 preferentially cleaves Lys63 linkages in polyubiquitinated chains containing both Lys48 and Lys63 linkages (Winborn et al., 2008). Overexpression of normal human Ataxin-3 can protect *Drosophila* from polyQ-huntingtin-induced neurodegeneration (Warrick et al., 2005). Unlike wild-type Ataxin-3, MJD-linked mutant Ataxin-3 can promote ubiquitination and autophagic degradation of parkin probably through regulating the levels of Lys27- and Lys29 ubiquitin-linked parkin (Durcan et al., 2011).

## Conclusion

Although UPS-mediated protein degradation has been well-studied in the last few decades, regulation of ubiquitination and proteasomal degradation in normal brain function and pathogenesis of neurodegenerative diseases remains largely unknown. The regulation of polyubiquitination processes may vary at different physiological and/or pathologic conditions. This review surveyed dysregulation of UPS in several major neurodegenerative diseases, more research is required to understand how the UPS is disrupted in neurodegenerative pathogenesis. By enriching our understanding of protein degradation, we can begin to formulate strategies to treat neurodegenerative diseases remediating aspects of UPS dysregulation.

## Author Contributions

QZ, LZ, and XW wrote the manuscript, TH, YZ, HL, and HX edited the manuscript. All authors read and approved the final manuscript.

## Conflict of Interest Statement

The authors declare that the research was conducted in the absence of any commercial or financial relationships that could be construed as a potential conflict of interest.
